# Valedictory Address before the Baltimore College

**Published:** 1847-03

**Authors:** Amos Westcott

**Affiliations:** Professor of Operative and Mechanical Dentistry.


THE AMERICAN

### Journal of Dental Science.

Vol. VII.] MARCH, 1847. [No. 3.

### ARTICLE I.

*Valedictory Address, delivered before the Graduating Class of the Baltimore College of Dental Surgery, for* 1846–47. By Amos Westcott, M. D., Professor of Operative and Mechanical Dentistry.

Gentlemen:

I have cheerfully embraced this occasion of addressing you, although, with the pleasure that this honor affords me, are mingled emotions of pain and diffidence: of pain, because our relation of teacher and pupil is now closed, and our pleasant connection, growing out of this relation, is about to be broken up; of diffidence, because it involves the responsibility of giving you parting advice.

You have now finished your collegiate course, and are about to engage in the active duties of your profession—a profession which involves many trials and responsibilities, and one which, as it is ill or well exercised, is to prove the bane or benefit of your fellow beings. In the selecting, therefore, a subject upon which to address you, I shall presume that one most interesting, which most concerns your future welfare, and is most intimately connected with your success in the practice of your profession.

Were it announced to you, on good authority, that an invention had been made, by which it was possible for you to convert into *gold*, all the commoner articles, which lie in great profusion around you, and which are at your command, would you not be interested in learning all that might be learned concerning such an *engine* of wealth and power?

And would not this be the case particularly, were you to learn, in the same connection, that its use was free to all, provided, simply, that each manufacture one for himself?

Upon such an announcement, what would be among the first inquiries that would come up in your minds, allowing you could be made to believe that the statements in regard to its powers were strictly true, and had, moreover, been the result of rigid and well tested experiment.

Would not the inquiry forthwith be raised in the mind of each of you, *is it possible for me to construct such an engine?* And would not an affirmative answer to this question settle all others? But suppose, on investigation, the construction were to require ten of the best years of your life, and, moreover, to involve a necessity for the exercise of the utmost industry, perseverance and economy; would all this deter you from the undertaking, allowing that you could be made certain of ultimate success?

I can anticipate your answer. You would, with one accord, exclaim, *give us the plan*, and we will most cheerfully embrace the undertaking.

Fully imbued with a certainty of success, you would despise all fatigue, and covet all hardships necessary to your advance. The plainest meal would become your richest dainty, if but seasoned with the belief that it was conducive to the consummation of this grand scheme.

Gentlemen, this discovery has actually been made, and the virtues of this engine have been fully tested, and its powers fully established!

*Its name is professional excellence.*

Therefore, to the attainment of professional excellence, as a subject in the highest degree worthy of your consideration, let me invite your attention.

The position which I shall assume, in the remarks I shall offer on this occasion, is, that *professional excellence is the only*

***sure guide which can conduct the professional man to eminence and fortune*** ; and that high attainment, combined with moral worth, are almost sure to possess him of both. I shall, therefore, hold them up as the prominent incentives to the acquirement of skill in the profession which you have chosen, and for your proficiency in which, you have now received your first honors.

In the discussion of this subject, I shall not attempt to show how far, *fame*, for its own sake, is a worthy and noble object of your ambition, or whether it should ever be sought, other than as a *means* of securing other objects, whose value is acknowledged by all. It would, indeed, be a most difficult task to isolate this, as a distinct object of pursuit, from its almost necessary accompaniments—wealth and power.

Nor does it matter whether we regard eminence or fame as a primary object of pursuit, or whether we consider it simply as the avenue through which goods more substantial are to be secured ; the fact that it has ever been the object of ardent solicitude and pursuit, remains the same. The love of military glory has marched its thousands and its tens of thousands on to the field of battle—into the very cannon's mouth ; the love of fame has called forth the best talents and energies of the statesman and the scholar, and, as the means of acquiring wealth and power, has ever been the strongest motive to human exertion.

That professional fame or eminence is the legitimate offspring of professional excellence, is a proposition which hardly needs illustration.

It is true, that fortuitous circumstances, or even intrigue, may sometimes give an individual a species of eminence ; but any elevation in professional rank, not based upon real attainments, is necessarily evanescent in its character.

We need not go out of our own profession for illustration. At several different times, the newspapers and periodicals have been loaded with wonderful discoveries, which, for the time, secured for the pretended discoverers a temporary fame, and a corresponding pecuniary reward. The astonishing virtues of the royal mineral succedaneum were, at one time, at once the wonder and admiration of the age ; and, at this very moment, the same news sheets are *delighting* the public, by announcing a discovery, which promises well nigh to remove the whole curse consequent upon the fall of Adam. We are now assured that *pain* is hereafter to be ranked with the things that *were*, and that, if agony itself is not a mere *humbug*, it is now so easily transformed into sensations of delight, that he who endures it, must do so from a mulish obstinacy.

Both of these discoveries have professedly in view, the same noble, and generous object, and from the astonishing virtues claimed for them, we might well expect that one more turn in the wheel of time would complete the great work of re-organizing the human constitution, on a plan, not admitting of the ills which flesh is now heir to. Such revelations are truly *god-sends*, alike to genius and the world. In the light of such examples well may we conclude that "the battle is not *always* to the strong, nor the race to the swift, nor riches to men of understanding."

But, alas, to tell the truth, the former of these splendid discoveries has *unfortunately* been exploded ; and the latter, to say the least, is made of very explosive materials—one of the most inflammable of *gaseous* compounds.

But notwithstanding these *imposing* examples of *etherial* fame, the general law is excellence before eminence. Read the history of those discoveries which have stood the test of time, and which have in fact proved great blessings to mankind, and you will find that they have been the result, almost without exception, of patient research, of careful analysis, and a rigid application of first principles, and of a long continued series of experiments, and have always been progressive in their development.

As an illustration, let us glance at the various discoveries connected with the subject of electricity. Without an examination into the progressive developments of this science, one would be led to infer that the application of electricity to the *telegraph*, which is very justly attracting so much attention, and which has so effectually annihilated both time and distance, in the transmission of intelligence, was the result of some sudden revelation, made by the mighty genius of the discoverer.

But how stands the fact? But for the unremitted, and protracted labors of *Franklin*, the nature of this subtle agent, and the laws by which it acts, would, probably, still have been, comparatively, unknown. Others commenced where Franklin left off, availing themselves of his discoveries, and by a similar application of untiring perseverance, developed new features of this interesting science. This agent was soon found capable of *creating* for its own purposes, a most powerful auxiliary, in the form of magnetism.

At length, by their combined agency, oscillatory motion was produced.

This accomplished, the firm foundation was laid for its application as a motive power. The next prominent achievement was the production of *rotary* motion, which was first accomplished by the far-famed Brandon *blacksmith*, who, by day, had plied his energies to his anvil, and, by night, to the study of science. You will see, then, that prior to the application of electricity to the telegraph, every *principle* which this invention involved had been discovered, and every *application* necessary to all the various kinds of motion had been made.

It is therefore plain, that the invention of the electro-magnetic telegraph not only involved a knowledge of all prior discoveries, but it is equally plain that it constituted but a single link, in the long and unbroken chain of discovery, connected with this most interesting subject.

If any doubt still remains in your minds, in respect to the basis of professional eminence, just call to mind all the different eminent men of your acquaintance, and compare their lives, with the stations they now occupy. Such a comparison can but result in a full conviction that excellence is the law of eminence.

Nor need you go out of our own profession for examples to establish this conclusion.

Who, then, will say that professional fame is the offspring of chance, or fortune; or, that it does not require time, and care, to bring it to maturity. If you are occasionally called upon to witness elevation, without a corresponding excellence—one, which, like Jonah's gourd, has grown in a night, you may reasonably suspect that it is spurious in its character, and that, like that same gourd, it will perish in a night.

Let us next consider professional excellence as the basis of *wealth.*

A good reputation is a thing of the highest value to the professional man, in a pecuniary point of view.

It is true, that every man, whose eminence in his profession is great, does not become rich, yet, it is nevertheless *certain*, that without reputation, no man ever accumulated wealth, rapidly, *by* his profession.

If the man of eminence does not accumulate wealth, it is not because he lacks the means of *getting* money, but, rather, because he has not the inclination or the faculty to keep it.

In this country there is a strong predisposition, on the part of young men, to get an early start in business; frequently, sadly to the neglect of those qualifications indispensable to success in after life. We often see apprentices leaving their masters with their trades half learned—students, in the different professions, exercising their utmost ingenuity, to secure an early examination, and a premature release from the bonds of studentship, as if a diploma, no matter how obtained, could, by some magic virtue, secure to them success. I hardly need add, that this is a most fatal mistake, especially at the present day, when titles are so cheap, and confer so little distinction.*

And I am sorry to say, that students in the dental profession, have been pre-eminently obnoxious to this charge of commencing the practice, and assuming the responsibility of their profession, while ignorant of its first principles—frequently endeavoring to make up by flaunting newspaper advertisements, and bombastic pretensions, what they lack in real excellence. This

* Professor Samuel Jackson, of the University of Pennsylvania, in speaking of the depressed standard, as regards the medical profession, observes: "The title of *Doctor* is ceasing to be a distinction. The only requisition is the *cash,* or the credit, for the cost of the tin plate, on which it is printed. The M. D. is fast sinking, and will probably soon reach the same ebb." "In this state of affairs," he very justly observes, *"individuals must rely on themselves for their success and reputation.*—Int. Lecture, Nov. 3, 1846. p. 14.

has been done, till it has become proverbial, that long advertisements, setting forth the peculiar, and extraordinary merits of the author, are a sure sign that these are a necessary substitute for merit; and they certainly are the very best *prima facie* evidence of quackery.

Now, I would, by no means, attribute dishonesty of motive, to all who thus cheat themselves, and, who, if they are employed, cheat their patrons. The chief difficulty is, that they have wholly misappreciated the nature, and extent of the subject, and the fact, that excellence alone, can secure permanent success. The profession has been regarded easy of access—as requiring but few accomplishments, and those soon acquired. This accounts, at once, for the rapid ingress into its ranks, and for the constant failures of success by those lured into it, by such groundless presumption. It is a notorious fact, that, the number of dentists in the United States was nearly doubled within the two years, following 1836. This is easily accounted for. The derangement of business which followed the general embarrassment of the country, during this period, threw thousands out of employ, and deprived them of situations. Now, many of these having imbibed the notion that dental surgery is easily acquired; and, moreover, looking upon it as a lucrative calling, had no hesitation in assuming the title of doctor, trusting to luck for such qualifications as *they* deemed essential, and never doubting that they were on the high road to wealth, at least.

These men having no skill on which to base success, became clamorous in pretension—each offering to *warrant* that he would do, *at a very low price*, that which no man else could do at all.

But have these adventurers reached the goal for which they started? Have they acquired either eminence or wealth? So far from this, they have generally failed to secure a competence, and, what is far worse, they have created for themselves a temptation to do what *they*, under other circumstances, would have despised—a temptation to secure, by culpable stratagem and misrepresentation, what would have been the legitimate result of integrity and professional excellence. An attempt, therefore, to secure, without excellence, what can only be attained by it, not merely results in disappointed hope, but often in the prostration of character and honesty. By means like these, our noble profession has been lowered, in the estimation of many, to a standard far below that of the most humble trade; to say nothing of the immense amount of suffering which has resulted from the malpractice of such operators.

But let us leave this side of the picture, which must be painful to every one possessed of common sensibility, and examine the opposite.

Call to mind those men who have made great reputations and splendid fortunes, by the legitimate exercise of their professions, and you will invariably find them such as have aimed at excellence, and have steadily pressed forward towards this prize.

If, as in some cases has been true, they were not as perfectly qualified at the outset as they could wish, they did not conclude that all progress was unnecessary, as soon as they could earn a mere pittance, by bringing into requisition the entire stock of their attainments. If they were obliged to avail themselves of the knowledge they possessed, the greatest possible share of their income was devoted to their farther advancement, and this system was continued, till all obstacles were overcome to excellence, eminence and wealth.

But, on the other hand, he who stops all progress towards excellence in his profession, so soon as he can make it earn him a dime, will soon find that he is obliged to content himself with earning pennies, while his industrious competitor, who started in his rear, but whose aim has been high, will be earning dollars. To let, therefore, the love of hoarding up money supersede a desire to excel in your profession, especially while you are young practitioners, is to be "a penny wise and a pound foolish."

First seek *excellence*, and eminence, then wealth, will naturally, I may almost say inevitably, follow.

Reverse this order, and neither will be secured—nay, poverty and obscurity will be a certain result, unless you strike from the legitimate path of the professional man.

Why do some practitioners command a large fee for their services, while others are obliged to content themselves with but a tithe of the amount? And why is it that those who receive these large sums, are constantly employed for a long series of years? And why do they receive patronage from the most remote portions of the country? Is it because those who pay such prices, and take such great pains to secure their services, do so to gratify a peculiar taste, independent of an expected equivalent? And have these eminent men acquired and retained this pre-eminence over their professional brethren by stealth or stratagem?

There may be instances where mere pretension has created a short-lived fame, and taken on all the livery of merit, and reaped, for a time, the reward due to excellence, but the ass who would thus parade in the lion's skin, is sure to be exposed, and well is it for him, if no tread-mill is at hand.

Allow me to mention another incentive to the acquirement of excellence in your profession. You have a higher motive than fame or gold to reach this standard, and it is the augmented ability *to do good*—to confer lasting favors upon those whom you serve.

The love of fame is a natural sentiment, and its attainment, by legitimate means, is strictly in accordance with the laws of our being.

The love of gain is also a clearly marked, natural and constant feeling, and the desire to acquire was, undoubtedly, implanted by the Creator in the mind of man, for wise and benevolent purposes, and the cultivation of any art or science, with reference to the "recompense of reward," infringes, of necessity, upon no law which we are called upon to observe.

But the Creator has implanted in the mind of man a higher and nobler sentiment, which derives its gratification from doing good to his fellow man, from dispensing happiness and comfort to those around him, and in alleviating or curing those ills which he cannot avert.

Here, gentlemen, is a field in which you may not only become legitimate, but prominent laborers.

It is the province of our profession to prevent and cure disease, and to alleviate sufferings. Its scope is not even limited to this. It is capable both of preventing and correcting *defor-*

*mity*—of restoring to pristine beauty the time-worn features; and of bringing back the youthful smile, to enlighten the countenance which has, by a premature destruction of the dental arch, been disfigured and depressed. Will not the ability to exercise your profession thus, be a source of gratification, unknown to the aspirant after mere fame and wealth? Will not the blessings invoked upon you by the thousands who may have received such benefits at your hands, be a source of consolation which will buoy you up under the fatigues and trials incident to the *faithful* exercise of your profession, and furnish you a powerful motive to master all its resources? The following anecdote, related by Dr. E. Parmly, of one Mr. Waite, formerly an eminent dentist of London, will serve to illustrate the pleasure of conscious excellence. Dr. P. says, "while I was residing in London, an elderly gentleman showed me a tooth, having in it a large and beautiful stopping, done by Mr. Waite, more than twenty years before, and informed me that when the operation was completed, Mr. Waite clasped his hands together and exclaimed, 'When you are an old man, you will look upon that tooth, and say *blessed be Waite.*'" Gentlemen, I envy the man who can truthfully make a similar exclamation in regard to every operation going from his hands. Gold is not the price of such emotions. Who, then, does not covet the reputation of doing good.

Surely such "a good name is rather to be chosen than great riches," and such "favor, rather than silver or gold." But whether you would seek fame, wealth, or the pleasures of beneficence, as the reward of your professional labors, remember, that excellence is the only sure basis upon which either can be built.

Any other foundation is of *sand*—liable, any moment, to be swept away.

The question has, doubtless, already come up in the mind of each of you, is it possible for *me* to acquire the excellence of which you speak, as being so desirable?—and, if so, how shall it be accomplished?

Let me then direct your attention to this part of our subject—*the means of acquiring excellence in your profession.*

Upon this point, it may be observed, that a correct appreciation of the value and necessity of excellence, accompanied with the firm resolve that you will secure it, is of more importance than any particular means—more, indeed, than all else beside. "He that has the will, will find a way," is a saying no less true than trite. I have already spoken of the value of reputation, and of high attainments, as the basis of success, and I may be permitted here to add, that at no period has this necessity been so great as at the present time. This enhanced necessity has been created, mainly, by the present astonishing facilities for rapid, easy, and cheap communication, which have virtually converted the whole country, into one vast community. Formerly, our competitors were our immediate neighbors, now they may be those residing hundreds of miles from us. Formerly, to ensure success, we had only to compare favorably with those in our immediate vicinity, now we have to compete with the *world.*

But should this be a matter of disparagement?—by no means. It should rather constitute the strongest incentive to put forth redoubled energy—one which should make your firm resolve, wax *firmer.* What advantages do others possess which are not within your reach? While the demand for extraordinary acquirement has increased, the *means* have much more abundantly increased. Compare the facilities which you may now enjoy, with those commanded by the men who have gone before you, whose names you venerate, and whose attainments you should emulate.

Instead of being doomed to find and keep the true path, by the dim light of your own experiments, without guide, or compass, you are now permitted to glide with the greatest rapidity, upon rail roads constructed by them, over territory, which, to these pioneers, was an unexplored wilderness. I therefore point you to the recorded experience of eminent men in the profession, as the first special means of acquiring excellence. In other words, study the different works which have been written upon dental surgery.

Books are the great repository of learning—the reservoirs, holding the accumulated experience of those who have preceded us, in the cultivation of the arts, sciences, and literature.

By availing yourselves of this source of information, you will learn by what means excellence has hitherto been acquired, and by what process, successful operations have been performed, and by what remedies diseases have been cured. In short, you will thus obtain, so far as books are capable of imparting this information, the combined experience of many of the most eminent men in our profession. But in a profession like ours, involving the nicest and most difficult *manipulations*, books can never wholly supply the place of *personal* instruction. More is frequently learned from the latter source, in a few minutes, than you could glean from books in days; if indeed it could be so obtained at all. You will, therefore, find it greatly to your interest to become *personally* acquainted with eminent men, and seek the instruction to be derived from this source. Formerly it was difficult to get access to the private practice of skilful practitioners. A feeling pervaded the profession, to a very great extent, that the skill which each possessed, was his private property—his capital in business, and, that, if he imparted it to his neighbor, he did so at a sacrifice. But I am happy to believe, nay, to *know*, that this selfish, and short-sighted feeling, is rapidly giving way to one more liberal, and more profitable to all parties.*

* The credit, to a very great extent, of working this change among the profession, is due to the American Society of Dental Surgeons. This body embraces a large number of the best practitioners of the country, and it being one of the leading objects of this association to cultivate the profession *by united effort*, this influence has been by no means confined to its own ranks, but has been felt and acknowledged far beyond the precincts of the Society. I think I may safely say, that if you present proper evidence that information, for noble ends, is your object, you may now be cordially entertained, by every member of the profession, whese counsel is worth your seeking, and I would most earnestly advise you to seek and cultivate the acquaintance of men who have themselves attained excellence and eminence. One of the leading objects gained by membership in the American Society, is the facility it gives of making profitable acquaintances.

Its annual meetings bring together more than you might otherwise meet in years. The Society's Journal, or the American Journal and Library of Dental Science, I would also recommend to your favorable notice. This is a periodical worth, to every practitioner of dental surgery, many times its cost. The Library part alone, which consists mainly of the republication of

As another special means of arriving at excellence in your profession, I shall recommend to you its study as a *science*—not merely learning facts and processes, but availing yourself of the principles upon which all the various operations and treatment depend.

Nothing is more common than to hear the greatest ignoramus vaunting about his *experience*, as opposed to science.

Now, while I would by no means undervalue enlightened experience, which is, indeed, the *basis* of science, I wish most firmly to impress upon your minds, that experience can never make up for the lack of science. The Chaldean shepherds might have watched the movements of the planetary hosts forever, and still have known less of the physical laws of the universe, than the mere schoolboy might learn, who pursues astronomy as a science, for a single week—nay, for a single day.

What did the experience of the ancient astronomers teach?—that the world we inhabit was the centre of a system, around which the sun, moon and stars revolved in their respective orbits. This whole scheme, which had been confirmed and consolidated by the *experience* of millions of observers, through successive ages, was demolished in one short hour by science.

What does experience teach us in relation to the constituents of our globe, and all the different objects belonging thereto? Experience, unaided by science, surely declares them to be innumerable and beyond comprehension.

She says to finite man, they are too extensive for your *inspection*, much more for your minute study. She declares, also, that the various observable phenomena of nature are hardly less extensive, and that the hope of comprehending them all, is a hope doomed to disappointment.

But let us interrogate *science*, and see whether she cannot the best foreign works, translated expressly for its pages, and which are not elsewhere to be found in the English language, would cost much more, allowing they could be procured, than the whole work as furnished by the Society. No practitioner should be without it, who can procure it, and any one in practice, who cannot afford five dollars a year, which is its annual subscription, for the only periodical of this character published in any country, may well conclude that he has mistaken his calling.

render the task less discouraging. Hear ye, and be astonished at her reply, when she declares that little more than fifty elementary substances compose the whole catalogue of created things; and that not more than one-half of this number is necessary to constitute all those commoner substances, with whose properties it is most essential for man to be acquainted!! Science makes another revelation, in the same connection, no less important and astonishing.

She declares that the *laws* by which these different elements combine to form the millions of sensible objects, are both few and simple! How perfectly, then, does the light of science change every feature of the picture!

Without science, the creation is composed of millions on millions of isolated objects; and the hardly less countless *phenomena* of nature, seem equally distinct and incomprehensible.

By the magic power of science, this mighty host of seeming entities, is resolved into a few simple substances, and all the phenomena arising from the operation of every law of creation, are correspondingly simplified, and even rendered subservient to the hand of man!

I must leave the application of these remarks to your professional pursuits, to be made mainly by yourselves. By a thorough perusal of the works of eminent men, by availing yourselves, as far as possible, of their personal counsels and instruction, and, by attending well to the *principles* upon which your operations and your prescriptions are based, you will possess advantages which will do much to aid you in your pursuit after excellence. But while it is highly proper that you should seek to avail yourselves of the experience of the past, and imitate those who have already reached the goal of eminence, I would warn you against indulging the hope, that you will be transported thither, solely by their efforts, or even by attaining the skill which they possessed. This is an age of improvement, and most emphatically such, as regards dental surgery; and hence, what would have been properly considered the *standard* of excellence in it, ten years ago, is by no means to be regarded as such at the present time.

Therefore, while you should be reading men, *you must be*

*working men.* In the profession which you have chosen, no kind of instruction whatever, can excuse you from actual and constant manipulation, and experiment, if you would excel as *operators.* Every dental office should, therefore, have a *laboratory* attached, with all the necessary apparatus for conducting experiments in every department of your science, and art. Be not content with the experiments of others, but perform them for yourselves.

By dividing your leisure between your library and your laboratory, with the firm determination that nothing shall remain hidden from your view, that can be discovered by untiring perseverance, you may well expect to reach a degree of excellence worthy the age in which you live. This course, moreover, should be adopted while you are young practitioners, for if you are successful, your leisure will gradually diminish, till none is left for such improvement.

But, gentlemen, I have presented the bright side of the picture, and I would that my duty required me to present no other. It is far more pleasant to dwell upon the means and chances of *success*, than upon the difficulties, the obstacles, and the temptations, lying in the way to high attainments. Were you to undertake the navigation of a particular river, it would concern you, not merely to know the character of the *bark*, best calculated for the enterprise, but, also, the character of the stream—whether there were not, beneath its smooth and placid surface, shoals and quicksands ; and, if so, where they were to be met with. It is no less necessary that you know the obstacles to, as well as the means of success, in order to be prepared to meet and overcome them.

Some of the intrinsic difficulties growing out of the *nature of the profession*,—I pointed out in my introductory lecture to the course which is now closed, and I shall now notice some of the more prominent, which are of an *extrinsic* character. These are divisible into two kinds.—*First*, those which are inherent in our own minds, and are alike obstacles in the way of high attainments in any pursuit, and,

*Second*, those dependant upon the present state of dental practice, and erroneous public opinion in regard to it.

Of the former class of obstacles, one of the most formidable, is *irresolution.*

This may originate from many different causes, and the remedy must vary accordingly.

A story is told of a lad who was found most unmercifully beating his own head, and, who, on being asked the reason of so unnatural a performance, replied, "*I am trying to raise my bump of combativeness*, that I may get courage to flog Jim ——, who has been imposing upon me."

Although I shall not recommend the exact means employed by this boy, to excite a given organ, or faculty, yet the example serves to present a general thought which is of great value—and it is this. You should study well your own faculties, and qualifications, relative to every inch of ground covered by the full extent of your profession, and seek by rigid discipline, and effort, to make up for any discovered deficiency.

If you find yourselves *irresolute*, seek the society of the *enthusiast*, in your profession, and by communing with him, learn to drink in his spirit and determination. Seek the society of those who excel you in their attainments, and ask them to impart to you the secret of progression. I know of no better cure for irresolution.

Again, you find yourselves reluctantly investigating, or practising some particular branch of your profession, evincing a distaste, or lack of relish, for this particular department. Here then is another "bump" that needs raising. How shall this be done?

I answer, *make agreeable*, *by habit*, *what is not so*, *naturally.* This *susceptibility* is one of the elementary constituents of our organization, both as regards body and mind, and is a provision, which pre-eminently exhibits the wisdom and goodness of the Creator—one which not only enables us to tolerate, but to enjoy, the various vicissitudes which would otherwise be insupportable. It is this susceptibility of acquiring fondness by habit, which makes the northerner love, and prefer, his native snow-clad hills; and, which makes this same individual, finally relish the sunny south, and shudder at the thoughts of return. It is this, which renders us capable of bearing up under poverty, and reverse of fortune. It is this principle which has given character to a majority of our *amateurs*, our *connoisseurs*, and our *epicures*, even in regard to those things and pursuits, for which they at first had no particular relish. This principle extends still farther, and makes *luxuries* of those things for which we have a strong natural repugnance. What human being ever had a natural relish for tobacco, opium, or alcohol? I will not inquire how many have come to regard these articles as luxuries, but rather how many have become positive slaves to them. I might continue to enumerate examples, both of mental and corporeal habits, where both neutral and repugnant subjects, and things, have not merely become tolerable, but indispensable, to what may be termed the artificial wants of habit; but I trust that enough have been presented to establish the principle, and to point you to the benefits which you may derive, by applying it to your professional pursuits. I beseech you, then, to regard nothing as permanently repugnant to your taste, which you have not tried to relish, and nothing which is valuable, as beyond your reach, before you have made a thorough trial to secure it. It is undoubtedly true, that a natural taste or bent of mind, is a great auxiliary to the *ready* attainment of excellence, in those matters, where it is applicable; yet, to conclude that a trial is useless, where no particular taste is at first perceived, or that *genius* is sufficient without industry, and effort is an assumption, not only contrary to all observation, but a mistake most fatal to improvement, and to the prospects of all who would secure excellence in any profession. The finest marble, unquaried, is out-shown by an inferior quality highly wrought. The rarest, and most beautiful varieties of wood, in the natural state, may not call forth one tithe of the admiration elicited by the more common ones, after they have been subjected to the hand of the skilful artist. The most magnificent engine, is useless, without a propelling power, while an inferior one may accomplish much, if energetically worked. So, genius, is neither admirable nor effectual, if suffered to lie dormant, while inferior talents, aided by energy and perseverance, may enable the possessor to attain to excellence, eminence, and wealth.

Beware, then, on the one hand, how you presume on *too*

*much genius*, and adopt the feeling that you need put forth but little effort; and on the other hand, that you possess *too little genius*, and, that, hence, no effort will be availing.

A false notion, in respect to genius, has cheated thousands out of eminence and fortune.

It only remains for me to notice the second class of obstacles referred to, or those growing out of the present state of dental practice, and erroneous public opinion concerning it.

Although this class embraces obstacles which are more tangible, and apparent, than those already named, yet, they are by no means the more real, or difficult to overcome. Supposing you have now paid all due attention, as *students*, to qualifying yourselves for the practice of your profession, it next concerns you to seek a location, and to secure a business. In the selection of your field, it would be natural for you to seek that which is most free from competitors, or, at least, where the ground was not occupied by men of high standing.

But were I to make, as the basis of advice, solely, your own interest, I should, by no means, advise you to seek a field where only *tares* had been sown, and where, hence, public opinion was arrayed against the whole fraternity. You would doubtless find far less to annoy you, in competing with high-minded, honorable men, upon the broad platform of merit, than with those who rely upon weapons which you would disdain to wield. But this advice must, of course, be limited, as, otherwise, good operators would be confined, comparatively, to a very few places. Indeed, wherever you may locate, you must be surrounded to a greater or less extent, by men, who are bold, and clamorous, in pretensions, and whose assurance is often a substitute for merit. But, however ignorant these men may be of their profession, you are not to suppose them ignorant of human nature, or of the available principles in the constitution of every mind.

They are, by no means, ignorant of the fact, that *every individual loves to save money, and to avoid pain.* These, hence, are selected as the assailable points; and, it is to these, that their batteries are principally directed. They, moreover, well understand, that *a strong desire is always favorable to, and, in many*

*cases, is capable of creating a faith in any promise, which proposes to gratify such a wish.* If, to these elements, you add a small quantity of *mystery,* and a little of the essential oil of some *patent,* as perfumery, you will have the *golden pill* which is so frequently offered to the public, and which they often swallow with too much avidity, especially, if accompanied with a few testimonials, no matter if from persons wholly unprepared to judge of the virtue of the article. That you may have more tangible evidence of the ingenuity, and skill, displayed in the compounding of these ingredients, I will call your attention to the following advertisements, wherein they all appear in their due proportions:—"Dr. ——— would specially urge those persons whose teeth have become so much decayed, as to be useless, to call and try *his improved method* of inserting, *whereby the necessity of extracting the old roots is obviated,* thereby rendering the whole operation of having *a complete set of perfect teeth,* that can be worn with ease, and may be used for masticating food, or in conversation, *with the same freedom as the natural* ones, inserted *without pain!!* * * * * * *

"☞*All operations performed in the evening, by gas-light, equally as well as by day-light!!*"

The following I clip from another paper: "Dr. —— has purchased Dr. Morton's patent, for using anodyne *gas,* by which *the patient may be put to sleep, and have the most painful operations performed without the slightest unpleasant sensation!!!* It perfectly obliterates pain, so that the patient will actually dream pleasant dreams during the operation, of having limbs amputated, teeth extracted, or any operation performed. * * * * Dr. —— removes teeth with *a patent instrument,* a great improvement from the usual system. He would remind his patrons that *he has practised dental surgery sixteen years—has a thorough knowledge of the medical profession,* and is well prepared to operate on the teeth in a neat, easy and *scientific* manner."

I simply give the caption to a third—which is as follows:

"Prices reduced!!—Teeth filled without pain! and without the use of the file!!"

The author of each of the above advertisements takes good care to inform the public, in the same connection, that all of these operations can be had at a very *cheap* rate. Nor do I hold these up as peculiar specimens, but rather as fair samples of dental advertisements at the present day. If you do not find these ingredients in exactly the same proportions in all, you will generally find them all to contain the same "*active principles.*"

Now, gentlemen, if the claims thus set forth are well founded, you are but poorly prepared to compete with this class of men—certainly not in their own way.

You have not been taught, at *this Institution*, that it is *admissible* to set whole sets of teeth on plate, with the dead roots left in the mouth, for the sake of saving the pain of extracting them. Nor that it is admissible to get your patient dead-drunk, either on *brandy* or *ether*, in order that they may avoid the pain attending extracting a tooth—nor that it is good policy to charge a price less than sufficient to secure *good* materials—nor that you could do justice to all cases of filling without the use of the file, nor yet that "gas-light" or "moon-shine" is a proper substitute for day-light, for conducting your operations.

Surrounded by, and being under the necessity of competing with, men making such pretensions, you will be subject to many temptations, but which may be mainly summed up in the two following:

1st. For the sake of securing business, to succumb to the wishes of your patients, in respect to the manner of performing your operations.

2d. To attempt to compete with them in their ruinously low prices.

Now, gentlemen, adopt either of these schemes, and your fate as professional men will be sealed. If you adopt the former, you will be obliged to subscribe to the latter—adopt both, and you may bid adieu to all hope of becoming either excellent or eminent. You cannot, as honest men, subscribe to the dictations of your patients, if their wishes, as is too often the case, are incompatible with correct practice; nor can you adopt a scale of prices below that which will compensate you for performing your operations *faithfully*, without at least creating for yourselves a strong temptation to make the operation correspond with the price.

To perform them imperfectly, for the sake of bringing them within a given circle of charges, is professional heresy—nay, downright dishonesty. There is but one safe rule upon the subject, and 'tis this—prepare yourself to judge correctly, and then suffer no temptation to drive you from the full exercise of your own decision. Kindly endeavor to convince your patient that your rules are in accordance with sound principles and enlightened experience; and this you are not to do by contrasting your own merits with your neighbor's faults, but by clearly exhibiting the principles upon which your operations are based. If, in this way, you fail to convince your patients, you can *respectfully* decline serving them, and that without making them your enemies. You may be firm, yet not uncourteous; plain, yet not rough; positive, yet not disrespectful. Again, adopt that scale of prices which will enable you to do all in the best possible manner. Temptations will gather thick enough, without *manufacturing* them, which all must do, who attempt to do business so low as to be unable to do it justice. But you will do far *better justice to all parties, to charge a fair price, and earn it.*

But, gentlemen, whatever you may charge, be faithful in your operations. Seek to make each one more perfect than the preceding, if by this course you sacrifice *money*—this you can far more easily regain than character. He who barters his reputation for money, kills, for the golden egg, the hen that laid it. But possibly you will say, these rules are too rigid, that one might starve before he could gain a stand by living up to them. *Then starve.* Better die a martyr to a noble cause, than live an empiric. But this cannot be the result of such a course of conduct. Is dental surgery such a profession, or is the world so blind, as that the faithful and honest operator cannot sustain himself without availing himself of the attractions of quackery?

Away with this hue and cry of *starve.* That your income at first will be small, I have no doubt, but there is an antidote for this.

The path through poverty is a hard beaten one, and has been graced by some of the greatest men the world ever knew. There are few, indeed, who have arisen to eminence, who have not known its way.

Let frugality, economy and patience be your attendants, and your journey will be both safe and easy.

In what way, then, are you to exercise economy to ensure success?

In supplying yourselves with *cheap* materials for performing your operations? by stinting your library? by using imperfect instruments? No, in none of these ways. You should never use any but the very best materials—better by far lie idle than do so. Make any sacrifice to make your library as complete as possible—in short, it is not economy to dispense with anything essential to the attainment of excellence, but it *is* economy to to dispense with every thing which does not contribute to the attainment of this cardinal object. I regret the necessity of so cursory a notice of this important topic, and, as a substitute for greater detail, I would recommend to you a thorough perusal of the life of Dr. Franklin, from which you will learn the true application of economy, and the results.

I cannot forbear giving you a single incident in the life of this illustrious statesman and philosopher, which is recorded by himself, and relates to a time when he was not only struggling against poverty, but was sorely opposed by competitors in business. Under these circumstances, he resorted to the following means to discourage his opponents: He sought out the leading man among them, and exhibited to him the engine upon which he relied to withstand their united battery.

If you have not read his life, you will be no less astonished than were his opponents, to learn that *it was simply the exhibition of his dining room in his back office*, where he had just finished his meal from a loaf of bread and a cup of cold water.

"Now," says Franklin to his competitor, "unless you can live cheaper than I, you must perceive that it is utterly vain to think of starving me out."

I need not say that his object was accomplished. Now which of you can claim to be better than Dr. Franklin, and yet which of you, for the sake of sustaining the honor of your profession, and promoting your own advancement, would be willing to live in your back office, upon bread and water? But till you have tried even this expedient, I hope none of you will think, for a moment, of deviating from the strictest rules of professional honor and integrity, in order to compete with the empiric.

Suppose Franklin, instead of the course which he adopted, had sought to overcome his opposition by *underbidding* his competitors, and to enable himself to do this, had done correspondingly poor work, what would have been the result? His name would probably have never gone out of Philadelphia, unless as "job printer." But Franklin was morally incapable of doing this mean thing.

In conclusion, I would say that I have reason for placing great confidence in the sentiments and rules I have now offered for your consideration; I can refer you to many an individual who commenced without a single farthing in his purse—the very sides of which seemed spasmodically collapsed—men whose extrinsic resources were nothing, and whose friends, like angels' visits, were few and far between, but who, by a rigid application of these rules, succeeded in gaining a high position and a lucrative business, under these discouraging circumstances. On the other hand, I could point you to a far greater number, who, by deviating from them, have been doomed to mediocrity, if not to poverty and perpetual obscurity. Reputation is of paramount importance to you as professional men, and permit me, in behalf of the faculty, to express our great solicitude for your advancement and success. May you add *yours*, to that catalogue of names, which will go down to posterity with all the honors due to excellence, to virtue and beneficence

It only remains for me to offer you the right hand of fellowship, as Doctors of Dental Surgery, and, in the name of the faculty, to bid you farewell

